# Long-term risk of a major cardiovascular event by apoB, apoA-1, and the apoB/apoA-1 ratio—Experience from the Swedish AMORIS cohort: A cohort study

**DOI:** 10.1371/journal.pmed.1003853

**Published:** 2021-12-01

**Authors:** Göran Walldius, Ulf de Faire, Lars Alfredsson, Karin Leander, Peter Westerholm, Håkan Malmström, Torbjörn Ivert, Niklas Hammar

**Affiliations:** 1 Institute of Environmental Medicine, Karolinska Institutet, Stockholm, Sweden; 2 Department of Medical Sciences, Uppsala University, Uppsala, Sweden; 3 Research & Development, Swedish Orphan Biovitrum, Stockholm, Sweden; 4 Department of Cardiothoracic Surgery, Karolinska University Hospital, Stockholm, Sweden; 5 Department of Molecular Medicine and Surgery, Karolinska Institutet, Stockholm, Sweden; Harvard Medical School, UNITED STATES

## Abstract

**Background:**

Elevated apolipoprotein B (apoB) and elevated apoB/apoA-1 ratio increase the risk of myocardial infarction (MI) and stroke, whereas high apoA-1 is protective. We study how these apolipoproteins are associated with major adverse cardiovascular events (MACEs), whether apoA-1 contributes to this association, and whether abnormal values occur decades before such events develop.

**Methods and findings:**

In the Swedish AMORIS (Apolipoprotein-related MOrtality RISk) cohort study, 137,100 men and women aged 25–84 years were followed an average 17.8 years. ApoB, apoA-1, and the apoB/apoA-1 ratio were analysed in relation to MACEs (non-fatal MI, stroke, and cardiovascular [CV] mortality), yielding 22,473 events. Hazard ratios (HRs) were estimated using Cox regression. Kaplan–Meier estimates were used to investigate the relationship of MACEs with increasing quintiles of the apoB/apoA-1 ratio in all age groups for both sexes. In nested case–control analyses, cases were randomly matched to age- and sex-matched controls, yielding population trajectories for apolipoproteins. Increased level of apoB and increased apoB/apoA-1 ratio were associated with risk of MACE and all clinical sub-components in both men and women across all ages (10th versus first decile in both sexes combined: HR 1.7 for MACE and 2.7 for non-fatal MI). Decreased values of apoA-1 potentiated the impact of apoB at all levels of apoB (on average across apoB range: 40% increase in HR for MACE and 72% increase in HR for non-fatal MI), indicating that the apoB/apoA-1 ratio covers a broader range of persons with dyslipidaemia at risk than apoB alone. In both men and women, MACEs occurred earlier on average for each increasing quintile of the apoB/apoA-1 ratio. Individuals with the highest levels of apoB/apoA-1 ratio experienced CV events on average several years earlier than those with lower ratios. Higher apoB/apoA-1 ratio in cases of MACE versus controls was seen already about 20 years before the event. A limitation of this study was that adjustment for tobacco smoking and hypertension was only possible in a small validation study.

**Conclusions:**

An imbalance between apoB and apoA-1 resulting in an increased apoB/apoA-1 ratio is strongly associated with the outcome MACE and its sub-components, in both men and women of all ages. An increased apoB/apoA-1 ratio already 2 decades before events calls for early recognition and primary prevention. Simple evidence-based cut values should be considered in future cardiovascular guidelines.

## Abstract 2

Göran Walldius and colleagues investigate the long-term risk of a major cardiovascular event by apoB, apoA-1 and apoB/apoA-1 ratio in the Swedish AMORIS cohort.

## Author summary

**Why was this study done?:**

- International guidelines for treatment of high cholesterol recommend monitoring of atherogenic low-density lipoprotein, non-high-density lipoprotein, and apolipoprotein B (apoB) for detection of cardiac risk. However, the protective effects of apolipoprotein A-1 (apoA-1) are not considered.
- In this long-term observational Swedish study, we measured the associations of apoB and apoA-1 with development of major adverse cardiovascular events (MACE).
- We wanted to see if the risk of MACE was associated to both apoB and apoA-1, summarised as the apoB/apoA-1 ratio.

**What did the researchers do and find?:**

- We linked apoB and apoA-1 laboratory analyses to clinical registers to investigate associations with risk of new MACE.
- Men and women of all ages were followed for decades, and 22,473 individuals had a MACE.
- We discovered that low protective levels of apoA-1 were associated with increased levels of apoB and a higher risk of MACE.
- In addition, a high apoB/apoA-1 ratio was strongly associated with the risk of having a MACE.
- In persons who had a MACE, the apoB/apoA-1 ratio was elevated already 2 decades before the event.

**What do these findings mean?:**

- Early detection and treatment of dyslipidaemia may prevent severe cardiovascular disease and save lives.

## Introduction

Dyslipidaemia is a major risk factor for development of cardiovascular (CV) atherosclerotic diseases. Low-density lipoprotein cholesterol (LDL-C) is considered the major causative lipid fraction, and lowering of LDL-C reduces the risk of CV events. LDL-C is therefore the preferred risk marker in international guidelines [1–3]. In newly updated guidelines, attention is also recommended for lipids other than high-density lipoprotein cholesterol (HDL-C) (non-HDL-C), as well as apolipoprotein B (apoB) in certain medical conditions [1–3]. ApoB also gained support as a risk marker in a UK Biobank study [4–6]. The potentially atherogenic cholesterol-containing lipoprotein particles very-low-density lipoprotein (VLDL), intermediate-density lipoprotein (IDL), and low-density lipoprotein (LDL) are transported in blood by apoB. The protective high-density lipoprotein (HDL) particles are transported by apoA-1. Both apoB and apoA-1 can penetrate the arterial wall. In various dyslipidaemias, both lipid-transporting processes may contribute to atherogenesis and increase the risk of clinical events, depending on the balance between these 2 major lipid-transporting processes [7–10].

We [10–14] and others [15–20] have shown that both apoB and apoA-1, and the balance of these apolipoproteins, expressed as the apoB/apoA-1 ratio, are strongly associated with myocardial infarction (MI) and stroke. However, in the latest international guidelines [1–3], apoA-1 and the apoB/apoA-1 ratio are not mentioned, but apoB is recommended as an alternative risk marker to LDL-C or non-HDL-C in individuals with type 2 diabetes and in patients with hypertriglyceridaemia or obesity [4–6]. The apoB/apoA-1 ratio gained further support as a risk marker in a meta-analysis based on 19 prospective studies [21]. The findings of the Emerging Risk Factors Collaboration meta-analysis of 36 prospective cohorts also give some support for the apoB/apoA-1 ratio as a predictor of CV risk [22], and large new prospective studies may be able to address this issue further [23].

In our present study of the Swedish AMORIS (Apolipoprotein-related MOrtality RISk) cohort, we investigate in detail the associations between apoB, apoA-1, and the apoB/apoA-1 ratio and the risk of a major CV adverse event (MACE) [11]. From follow-up of mean 17.8 years’ duration in 137,100 men and women aged 25–84 years at inclusion, we report CV events, including MACE, non-fatal MI, ischaemic stroke, CV death, and need of coronary intervention. We also investigate if apolipoproteins were elevated or abnormal already long before these events occurred.

## Materials and methods

### Study population

We used prospectively gathered data from the AMORIS cohort, consisting of individuals undergoing health examinations during 1985–1996 (baseline period) [7,11]. The cohort includes 812,073 men (49%) and women (51%) of all ages who were either healthy individuals taking part in routine yearly health check-ups through occupational healthcare or outpatients referred for laboratory testing. No individuals were inpatients at the time of the health examination. All individuals in the AMORIS cohort were residents of Sweden and predominantly living in Stockholm County (67%) at the time of their health examination. The total population of Stockholm County was about 1.6 million during the baseline period, of which the AMORIS cohort constituted a substantial part. A detailed cohort description is available elsewhere [7,11].

### Inclusion and exclusion criteria

For the present study we included men and women 25–84 years old without a history of MACE (non-fatal MI or stroke or CV death) or coronary artery bypass grafting (CABG) or percutaneous coronary intervention (PCI). All persons had information on apoB, apoA-1, total cholesterol (TC), triglycerides (TGs), and serum glucose from the same health examination (*n* = 137,100). ApoB and apoA-1 were part of an additional service from the laboratory on top of a standard blood analysis and applied broadly to unselected patients.

For this cohort we had limited information on the major CV risk factors tobacco smoking and hypertension. In order to take these risk factors into account, sensitivity analyses were performed based on persons included in the Work, Lipids and Fibrinogen (WOLF) study [24] and the Stockholm Cohort of 60-year-olds (60YO) [25] who fulfilled the study inclusion criteria (*n* = 13,636). Of these persons, 1,466 were also part of the AMORIS cohort (1.1%).

### Blood sampling and laboratory analyses

All blood samples were analysed as fresh blood at the CALAB laboratory, Stockholm, Sweden, between 1985 and 1996. ApoB and apoA-1 were determined by an immunoturbidimetric method according to Riepponen using polyclonal antisera from Orion Diagnostica (commercial assay), and reagents were always from the same manufacturer participating in the WHO/IFCC standardization program. TC was determined with the cholesterol oxidase/peroxidase (CHOD-PAP) assay, and TGs with the glycerol phosphate oxidase/peroxidase (GPO-PAP) assay, using enzymatic methods, with reagents from Boehringer Mannheim for the PRISMA instrument and from Bayer Diagnostics for the DAX instrument. All 4 methods were from the outset highly automated; from 1985 to 1992, the Multichannel AutoChemist-PRISMA (NewClinicon) was used, and from 1993 to 1996, the Multichannel DAX 96 (Technicon/Bayer) was used. All analysers were computerised with systems for automatic calibration. LDL-C was derived through lipoproteins using the Jungner formula [7] or the Friedewald formula [26]. The coefficient of variation was <5% for all laboratory tests. It should be pointed out that AutoChemist was originally developed at the CALAB laboratory by Gunnar and Ingmar Jungner. They also founded the CALAB laboratory in the 1960s.

Participants had their first complete profile of apoB, apoA-1, TC, and TGs done simultaneously. LDL-C and HDL-C were calculated by the Jungner formula. Concentration of LDL-C was calculated (panel A) based on concentrations of TC, TGs, and apoA-1 in 4,448 individuals recruited outside the present AMORIS population; concentration of HDL-C was derived from the Jungner formula: panel A, LDL-C = 0.48 + 0.99TC − 0.23TGs − 1.58apoA-1; panel B, HDL-C = TC − 0.45TGs − LDL-C. Concentrations of HDL-C were also measured by an automated precipitation method (Boehringer Mannheim, Mannheim, Germany) in a subset of individuals. Concentration of LDL-C was also calculated according to the method of Friedewald and colleagues [7].

### Co-variates

Type 2 diabetes at baseline was defined as present if the person was listed in the National Diabetes Register, if an ICD code corresponding to diabetes was present in the National Patient Register, and if the age at first diagnosis was >35 years, or if serum glucose at the baseline health examination was >7.0 mmol/L (fasting) or >11.1 mmol/L (non-fasting).

In the WOLF and 60YO cohorts, hypertension was defined as a systolic blood pressure > 140 mm Hg or a diastolic blood pressure > 90 mm Hg or self-reported hypertension [24,25]. Diabetes and smoking habits were self-reported, and ever tobacco smoking was defined as current or former regular smoking for at least 1 year.

Socioeconomic status (SES) was classified as blue-collar or white-collar work based on information on occupation retrieved from the population and housing censuses 1970–1990.

Record linkage to all external registers and databases used the unique Swedish personal identification number. Additional information on the definitions of BMI and co-morbidity is given in S1 Supplement.

### Outcome ascertainment and follow-up

New cases of MACE and its sub-components (non-fatal MI, stroke, and CV death) as well as CABG or PCI were ascertained throughout the follow-up period by record linkage to the National Patient Register and the National Cause of Death Register. The Cause of Death Register contains data regarding the causes of death of all Swedish residents, including if the person died abroad. ICD codes for all CV diagnoses are given in S1 Supplement. Persons were followed from the baseline health examination until the time of event, emigration, death, or end of follow-up on December 31, 2011, whichever came first.

### Statistical analyses

Baseline characteristics were described using means with standard deviations and proportions. Survival analysis using Kaplan–Maier estimates was performed for MACE by sex and age groups. Cox proportional hazards models with attained age as time scale were used to estimate hazard ratios (HRs) with 95% confidence intervals for development of MACE and its sub-components for quintiles or deciles of the apoB/apoA-1 ratio with adjustment for sex, TC, TGs, serum glucose, and SES. Corresponding analyses were also carried out by sex and age group. In the sensitivity analyses using the WOLF and 60YO cohorts, HRs of MACE and its sub-components were estimated by quintiles of the apoB/apoA-1 ratio adjusting for the above plus hypertension, tobacco smoking, and self-reported diabetes. A sub-cohort consisting of persons with baseline health examinations from routine health check-ups from the AMORIS study population (*n* = 39,007) was included in the sensitivity analyses to increase comparability to the WOLF and 60YO cohorts.

To evaluate the sensitivity of the apoB/apoA-1 ratio, apoB, and LDL-C for identification of cases of MACE and its sub-components as well as coronary interventions, the area under the receiver operating characteristic curve (ROC-AUC) was calculated. ROC-AUC values are compared using an algorithm suggested by DeLong, DeLong, and Clarke-Pearson [27].

### Nested case–control study

To describe trajectories of apolipoproteins preceding a MACE, MI, or CABG/PCI, we performed 3 nested case–control studies. Cases included all persons experiencing a CV event as defined above (MACE, non-fatal MI, or coronary intervention) during the study period. Incidence density sampling was used to randomly select 5 control persons per case from the cohort population at risk. Controls were matched to cases according to age (5-year cohorts), sex, and calendar year of the event. For MACE we had 22,473 cases and 112,252 controls, for MI 10,933 cases and 54,645 controls, and for CABG or PCI 7,777 cases and 38,885 controls. We generated trajectories of mean values of apolipoproteins in cases and controls separately, starting 25 years prior to diagnosis of the case or selection as a control person. These trajectories constitute a comparison between cases and controls at given time points and do not represent repeat measurements in the same individual. They do not represent individual development over time, but the mean values of cases and controls each year prior to diagnosis of the case or selection as a control person. This may be designated a ‘population trajectory’ [28,29].

Statistical analyses were conducted using Stata version 14.2 (StataCorp, College Station, Texas, US).

### Ethical approval, STROBE reporting, and funding source

The Swedish Ethical Review Authority (previously the Regional Ethics Review Board in Stockholm) approved the study (Record numbers 2010/1:7 and 2010/1047-31/1).

This study is reported as per the Strengthening the Reporting of Observational Studies in Epidemiology (STROBE) guideline (S1 Checklist).

The study was funded by the Gunnar and Ingmar Jungner Foundation for Laboratory Medicine, Stockholm, Sweden. The funding source contributed all laboratory measurements but had no other influence on the study.

## Results

### Study population characteristics

From the AMORIS cohort (Table 1) we included 137,100 persons fulfilling the inclusion criteria, of whom 43% were women. Mean age at inclusion was 48.9 years, and mean follow-up time was 17.8 years, generating 2,442,164 person-years at risk. The total number of MACEs was 22,473, with 8,567 non-fatal MI, 8,194 non-fatal strokes, and 5,712 CV deaths. Mean baseline values for total lipids, lipoproteins, and apolipoproteins were in the upper normal range. About half the population were blue-collar workers. Very few persons had a history of malignancy, kidney failure, or chronic obstructive pulmonary disease/asthma at baseline, 3.6% had diabetes, and 11% had a Charlson Comorbidity Index of at least 1. BMI was available for 28,613 persons, with an average value of 24.6 kg/m^2^.

**Table 1:** Person characteristics and follow-up time and event rates for MACEs.

Variable	AMORIS cohort			WOLF/60YO cohorts
	All	Men	Women	
Number of persons	137,100	78,199	58,901	13,636
Person-years at risk	2,442,164	1,378,168	1,063,996	198,715
Follow-up time (years), mean	17.8	17.6	18.1	14.6
Attained age at inclusion (years), mean	48.9	47.7	50.5	48.4
Attained age at exit (years), mean	66.7	65.3	68.6	63.0
Attained age at event (years), mean	71.2	68.3	76.1	64.4
MACEs, *n* (%)	22,473 (16.4)	13,988 (17.9)	8,485 (14.4)	1,025 (7.5)
Non-fatal myocardial infarction	8,567 (38.1)	5,930 (42.4)	2,637 (31.1)	462 (45.1)
Non-fatal stroke	8,194 (36.5)	4,797 (34.3)	3,397 (40.0)	399 (38.9)
Cardiovascular death	5,712 (25.4)	3,261 (23.3)	2,451 (28.9)	164 (16.0)
Women, percent	43.0	—	100.0	37.3
Blue-collar work, percent	52.1	43.5	63.6	56.9
apolipoprotein B, mean (SD)^1^	1.26 (0.36)	1.31 (0.35)	1.19 (0.35)	1.09 (0.30)
apolipoprotein A-1, mean (SD)^1^	1.43 (0.24)	1.37 (0.21)	1.51 (0.24)	1.41 (0.24)
apoB/apoA-1, mean (SD)	0.91 (0.31)	0.98 (0.31)	0.81 (0.28)	0.80 (0.26)
Total cholesterol, mean (SD)^2^	5.88 (1.18)	5.89 (1.15)	5.87 (1.22)	5.67 (1.11)^3^
LDL-C Friedewald, mean (SD)^2^	3.83 (1.10)	3.89 (1.07)	3.76 (1.14)	3.60 (1.00)^4^
HDL-C, mean (SD)^2^	1.52 (0.44)	1.37 (0.36)	1.70 (0.46)	1.48 (0.39)
LDL-C Jungner, mean (SD)^2^	3.71 (1.08)	3.78 (1.04)	3.62 (1.11)	3.56 (0.99)
HDL-C Jungner, mean (SD)^2^	1.58 (0.40)	1.46 (0.36)	1.73 (0.41)	1.50 (0.44)
Triglycerides, mean (SD)^2^	1.43 (1.09)	1.62 (1.22)	1.19 (0.81)	1.35 (0.95)
Glucose, mean (SD)^2^	5.10 (1.37)	5.20 (1.44)	4.96 (1.24)	5.35 (1.24)
Malignant neoplasms, percent	2.5	1.6	3.7	2.3
Kidney failure, percent	0.02	0.03	0.02	0.04
Charlson Comorbidity Index score ≥ 1, percent	11.1	11.2	11.0	11.3
COPD/Asthma, percent	0.9	0.7	1.1	1.0
BMI (kg/m^2^), mean (SD)	24.6 (3.8)^5^	25.4 (3.4)	23.7 (4.1)	25.8 (3.9)
Hypertension, percent	n.a.	n.a.	n.a.	22.3
Diabetes, percent	3.6	4.2	2.9	2.4
Smoking, percent	n.a.	n.a.	n.a.	54.0

^1^g/L.

^2^mmol/L.

^3^*n* = 4,200.

^4^*n* = 13,340.

^5^*n* = 28,613.

60YO, Cohort of 60-year-olds; COPD, chronic obstructive pulmonary disease; HDL-C, high-density lipoprotein cholesterol; LDL-C, low-density lipoprotein cholesterol; MACE, major adverse cardiovascular event; n.a., not available; WOLF, Work, Lipids and Fibrinogen.

The WOLF and 60YO cohorts (Table 1) consisted of 13,636 persons, of whom 37.3% were women, and mean age at inclusion was 48.4 years (WOLF cohort, 43 years; 60YO cohort, 60 years). A mean follow-up of 14.6 years generated 198,715 person-years at risk and 1,025 MACEs. The lipid, apolipoprotein, and glucose levels were similar to those in the AMORIS cohort, as was the frequency of co-morbidity. In all, 22.3% had hypertension, and 54.0% were ever smokers at baseline.

Further details on person-years at risk and frequency of MACE and its sub-components by sex and age group are presented in S2 Supplement.

### Kaplan–Meier estimates of risk of MACE in relation to quintiles of the apoB/apoA-1 ratio

**Fig 1:**
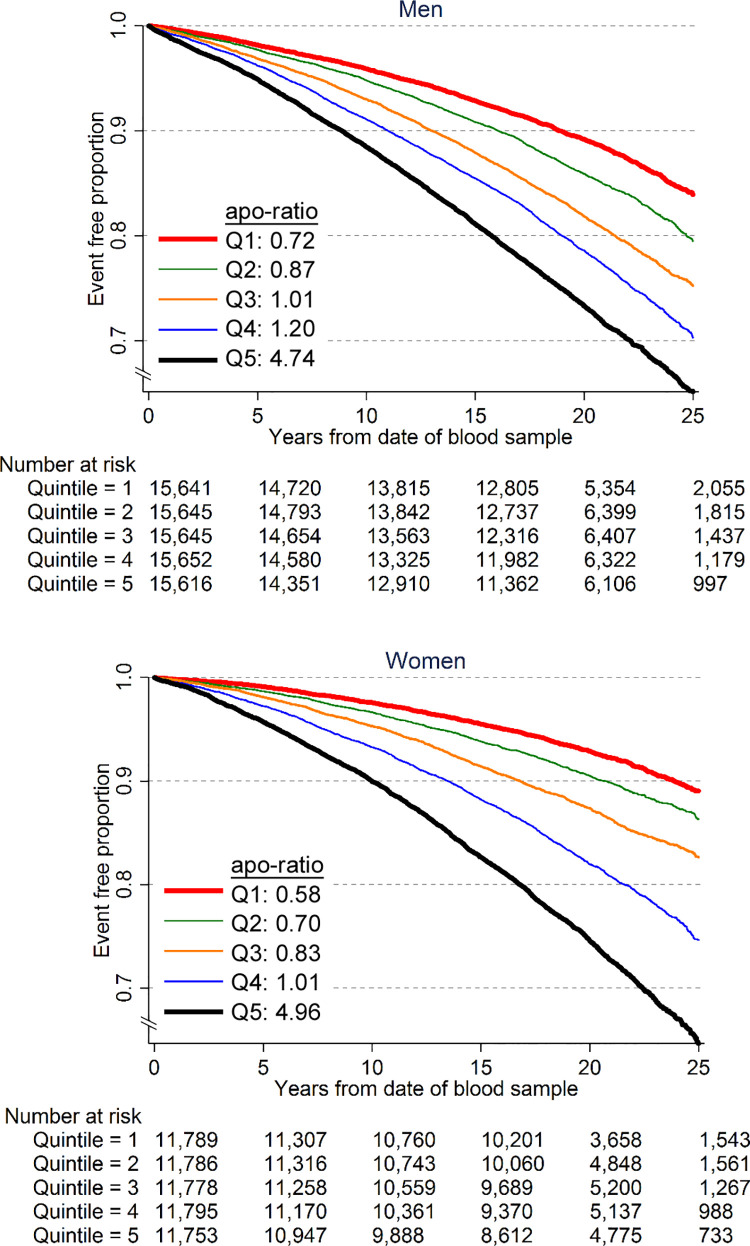
Kaplan–Meier estimate for quintiles (Q1–Q5, highest) of the apoB/apoA-1 ratio (apo-ratio) for major adverse cardiovascular events in men and women.

In both men and women, a MACE occurred on average earlier for each increasing quintile of the apoB/apoA-1 ratio at baseline (Fig 1). Among men, the time point at which 10% of individuals had experienced a MACE occurred at 9 years of follow-up in the fifth quintile, compared to 18 years in the first quintile. Among women, the corresponding difference was 10 versus 24 years. In corresponding analyses by age group, these differences tended to be even more pronounced in those up to 64 years of age (S3 and S4 Supplements). For example, in the age group 45–54 years, this difference was 9 years among men (19 versus 10 years) and 7 years among women (25 versus 18 years), while in the age group 65–74 years, it was 2 years among men (4 versus 2 years) and 3 years among women (8 versus 5 years).

### Hazard ratios for MACE and its sub-components

**Fig 2:**
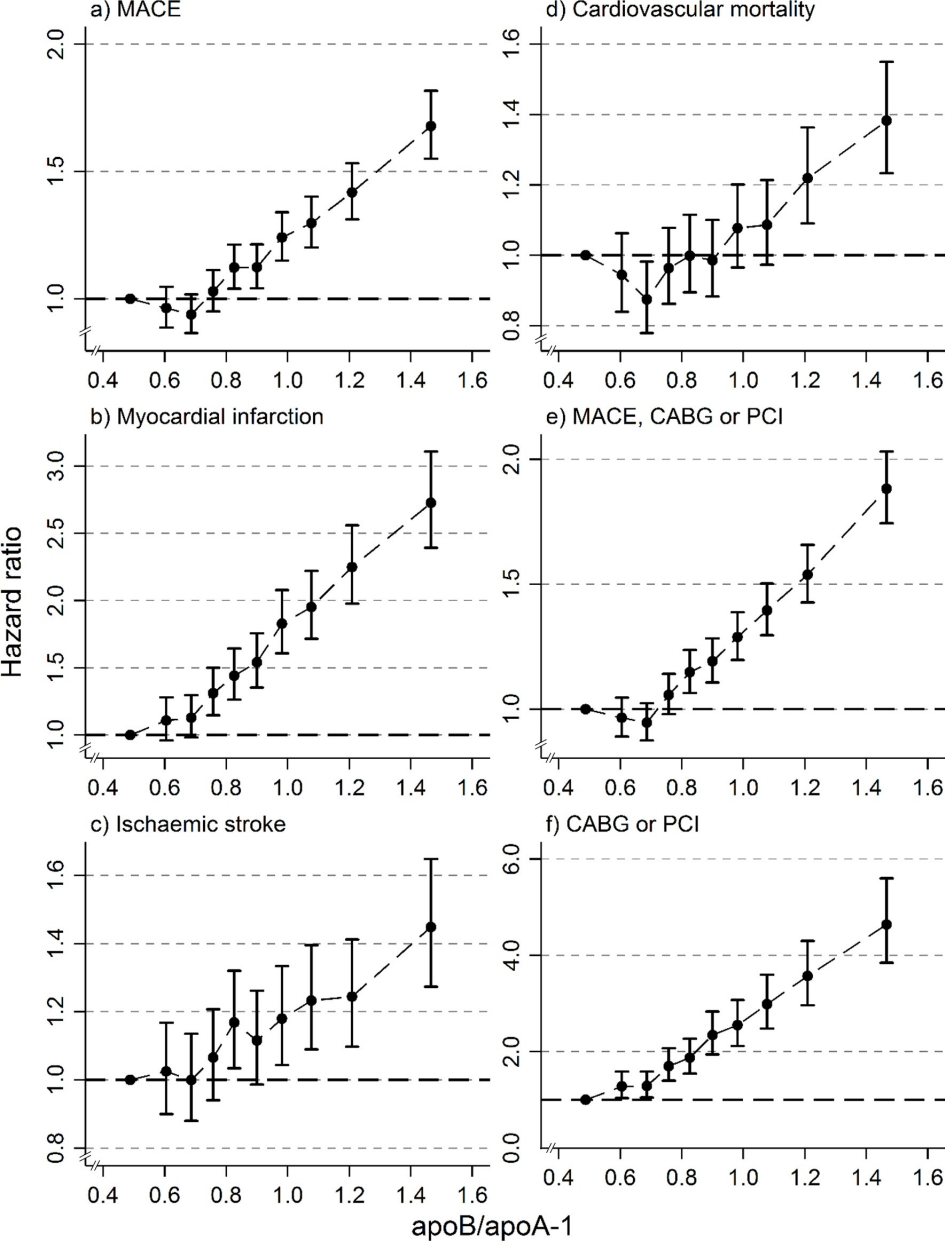
Hazard ratios of cardiovascular outcomes, with 95% confidence intervals, for deciles of the apoB/apoA-1 ratio. Men and women combined. Adjusted for total cholesterol, triglycerides, glucose, sex, and socioeconomic status. Outcomes: (a) major adverse cardiovascular event (MACE); (b) myocardial infarction; (c) ischaemic stroke; (d) cardiovascular mortality; (e) MACE, coronary artery bypass grafting (CABG), or percutaneous coronary intervention (PCI); (f) CABG or PCI.

There was a gradual increase in HRs with increasing deciles of the apoB/apoA-1 ratio for MACE and all its sub-components and CABG/PCI (Fig 2). For MACE, for apoB/apoA-1 ratios of about 0.8 and above, the HR increased gradually with increasing apoB/apoA-1 ratio, to a HR of 1.7 in the 10th decile. The increase was more pronounced for MI, with a HR of 2.7 in the 10th decile, and even more in those with coronary interventions, where, for apoB/apoA-1 ratios of 0.6 and above, the HR increased to 4.6 in the 10th decile. A graded increase with increased apoB/apoA-1 ratio was also found for ischaemic stroke, CABG or PCI, CV mortality, and MACE and coronary intervention as a combined event.

In S5 and S6 Supplements, corresponding results are presented for men and women separately. For MACE and all sub-components, as well as for coronary interventions, the findings were similar in both sexes as in the analyses presented in Fig 2.

**Fig 3:**
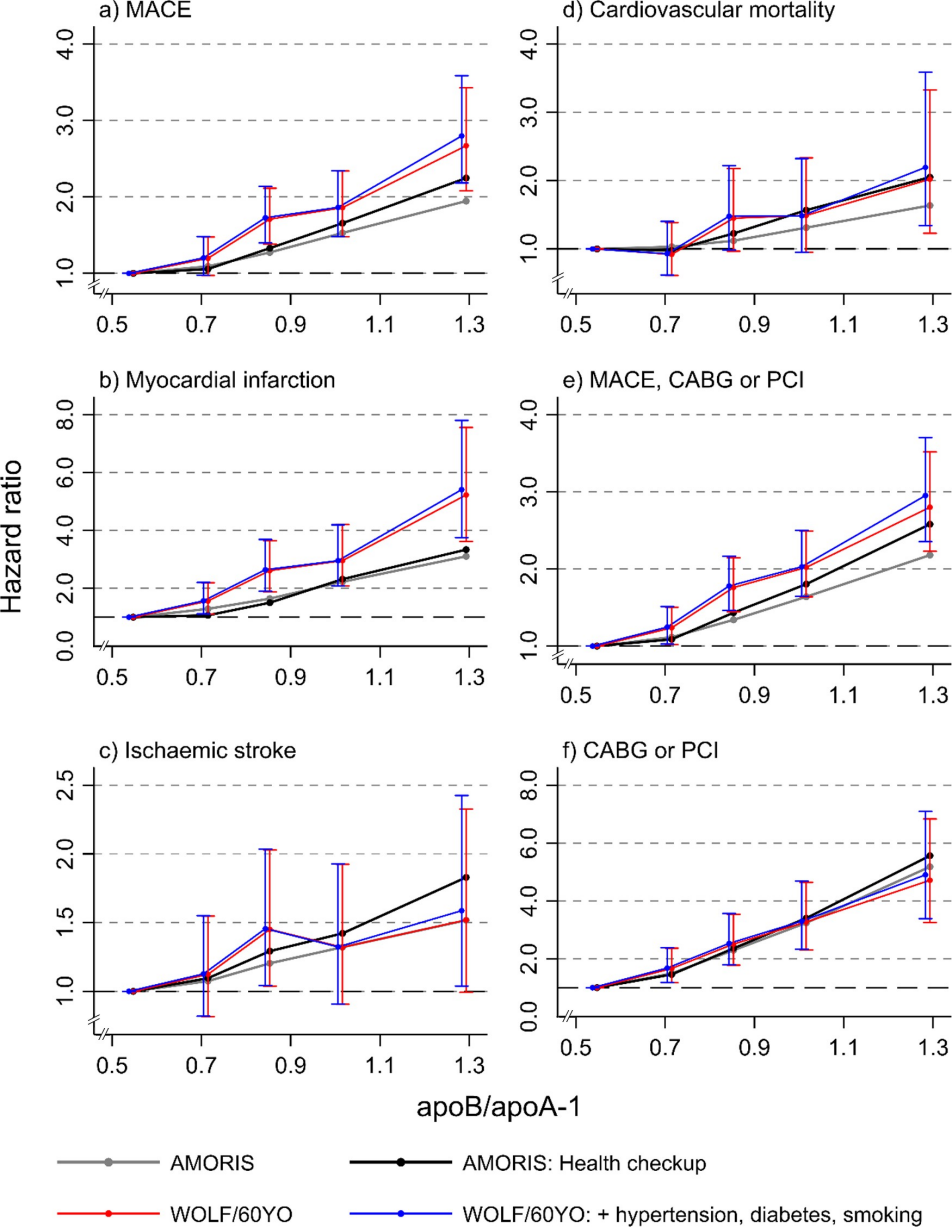
Hazard ratios of cardiovascular outcomes in AMORIS and WOLF/60YO, with 95% confidence intervals, for quintiles of the apoB/apoA-1 ratio. Men and women combined. Adjusted for total cholesterol, triglycerides, glucose, sex, and SES and, as noted in the figure, for hypertension, diabetes, and smoking. AMORIS: *n* = 137,100 (health check-up, *n* = 39,007); WOLF/60YO: *n* = 13,636. To enhance readability, the blue confidence intervals have been slightly shifted to the left, so as not to overlap with the red intervals. Outcomes: (a) MACE; (b) myocardial infarction; (c) ischaemic stroke; (d) cardiovascular mortality; (e) MACE, CABG, or PCI; (f) CABG or PCI. 60YO, Cohort of 60-year-olds; CABG, coronary artery bypass grafting; MACE, major adverse cardiovascular event; PCI, percutaneous coronary intervention; WOLF, Work, Lipids and Fibrinogen.

In Fig 3, the results of the sensitivity analyses based on the WOLF and 60YO cohorts are shown, adjusting for TC, TGs, glucose, SES, hypertension, diabetes, and tobacco smoking. Overall, the findings were like those for the total AMORIS cohort and for the sub-cohort from AMORIS of persons included through routine health screening. The additional adjustments in the WOLF and 60YO cohorts for hypertension, diabetes, and tobacco smoking had no substantial impact on the HRs.

### The apoB/apoA-1 ratio and its relations to levels of apoB and apoA-1—the ‘risk pyramid’

**Fig 4:**
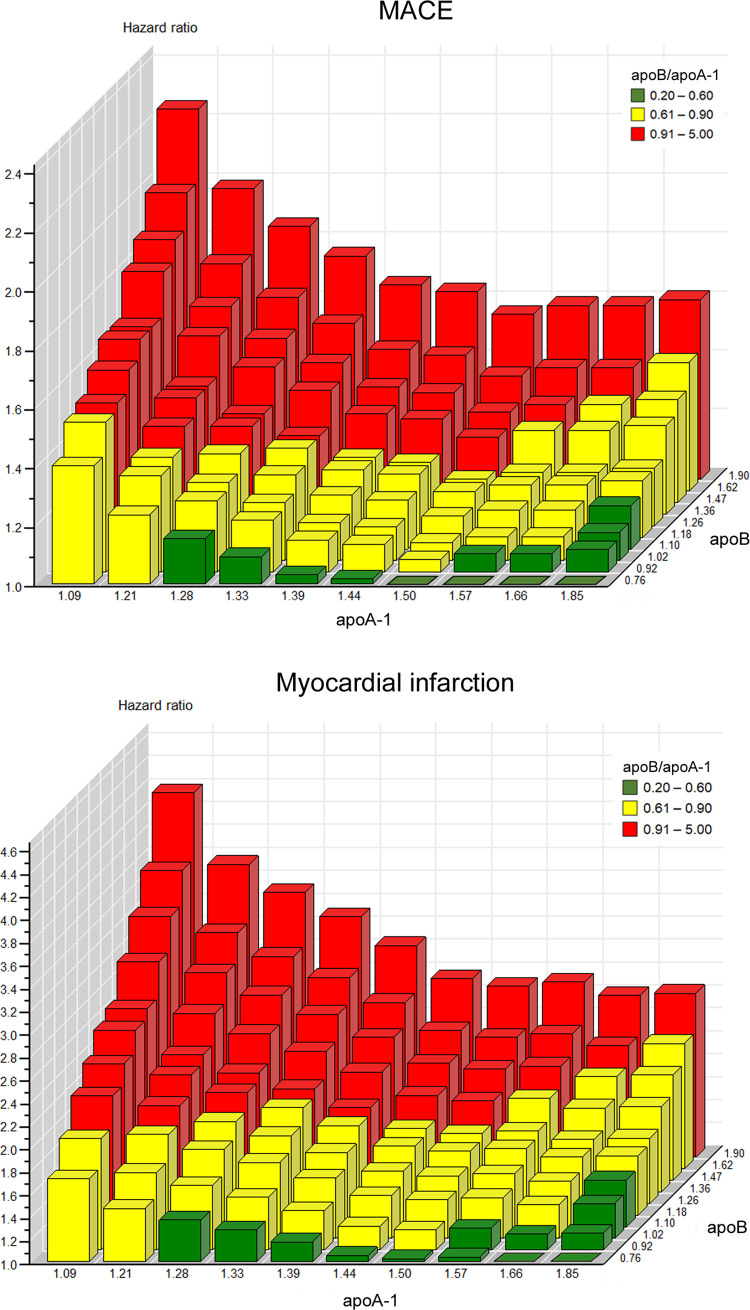
Hazard ratios of major adverse cardiovascular event (MACE) and myocardial infarction by the combination of deciles for apoA-1 (g/L) and apoB (g/L). Men and women combined. Adjusted for total cholesterol, triglycerides, glucose, sex, and socioeconomic status. MACE, top; myocardial infarction, bottom.

In Fig 4, HRs for combinations of deciles of apoB and apoA-1 are presented for MACE and MI. Three tentative levels of risk associated with the apoB/apoA-1 ratio are shown: low risk, 0.2–0.6 (green); medium risk, 0.61–0.9 (yellow); and high risk, 0.91–5.0 (red). The figure shows 100 different phenotype combinations of apoB and apoA-1 that form different levels of the apoB/apoA-1 ratio. The reference group was defined as the lowest decile of apoB and highest decile of apoA-1. For both MACE and MI, the pattern was of similar ‘pyramidal’ shape. The higher the apoB level and the lower the apoA-1 level, the higher was the HR associated with the apoB/apoA-1 ratio. The HR in for the MACE outcome increased at different levels of apoB by an average of 40% from the highest to the lowest apoA-1 values. For MI, the corresponding average increase of the HR was 72%.

A similar ‘risk pyramid’ indicating that low apoA-1 levels potentiate the HR associated with the apoB/apoA-1 ratio at all levels of apoB was found for all sub-components of MACE and for CABG/PCI, where the increase of the HR was on average 160% (S7 Supplement).

### ROC-AUC analyses

**Fig 5:**
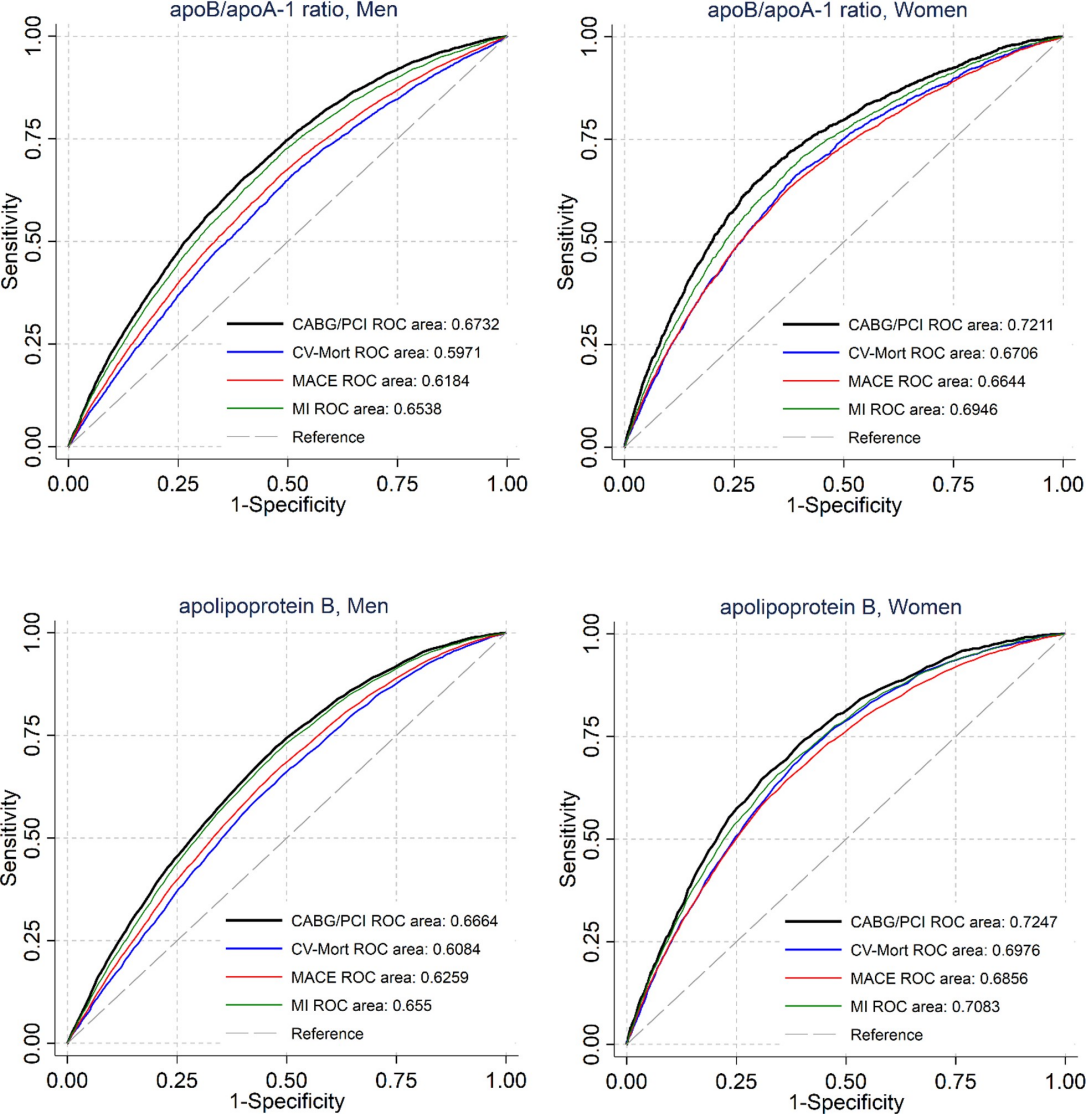
ROC-AUC for the apoB/apoA-1 ratio and apoB for different outcomes for men and women aged 25–69 years. CABG, coronary artery bypass grafting; CV-Mort, cardiovascular mortality; MACE, major adverse cardiovascular event; MI, myocardial infarction; PCI, percutaneous coronary intervention; ROC, receiver operating characteristic curve.

The MACE ROC-AUC for the apoB/apoA-1 ratio in persons aged 25–69 years was greater in women (0.66) than in men (0.62) (Fig 5). The corresponding ROC-AUC values for MI showed a similar sex difference. Women also had higher ROC-AUC for CV mortality, and the highest ROC-AUC was seen in for the CABG/PCI outcome. All differences were statistically significant (*p* < 0.0001). Corresponding analyses for apoB yielded similar results, with somewhat greater ROC-AUC values in women than in men for MACE, MI, and CABG/PCI.

In S8 Supplement, the ROC-AUC values are presented for apoB and LDL (calculated by the Friedewald formula, *n =* 18,069) for MACE and MI by sex. In this sub-population, the ROC-AUC for apoB and for LDL was greater in women than in men for MACE (apoB, 0.67 versus 0.61; LDL, 0.66 versus 0.60) as well as for MI (apoB, 0.73 versus 0.64; LDL, 0.71 versus 0.63). All these differences were statistically significant (*p =* 0.0001). The ROC-AUC for apoB versus LDL in the combined men and women group for MACE was 0.64 versus 0.62 (*p =* 0.0066) and for MI was 0.68 versus 0.65 (*p =* 0.0007). In analyses of apoB versus LDL by sex, a difference was seen only for MI in women (*p =* 0.0424).

### Trajectories of apolipoproteins in relation to time to MACE and MI

**Fig 6:**
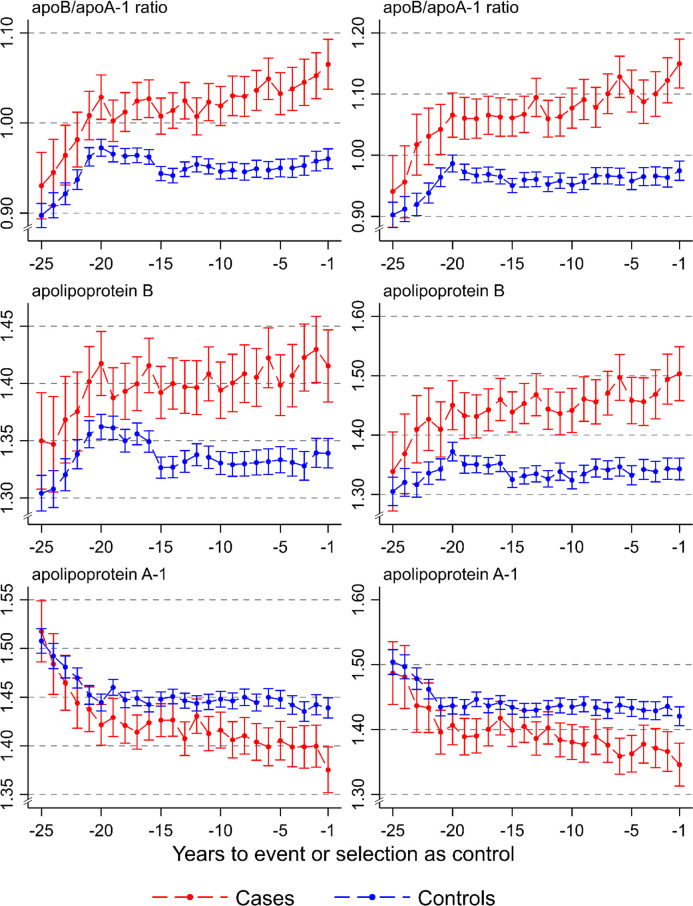
Trajectories of apoB/apoA-1 ratio, apoB, and apoA-1 over a 25-year period prior to a major adverse cardiovascular event (MACE) or myocardial infarction, for cases and controls, in men and women combined. MACE (left); myocardial infarction (right). ApoB/apoA-1 ratio (top), apoB (middle), and apoA-1 (g/L) (bottom). Note the different scales (*y-*axis) for the levels of the apolipoproteins (g/L).

In Fig 6, trajectories for the apoB/apoA-1 ratio, apoB, and apoA-1 over a 25-year period prior to a MACE or MI event are shown for cases versus matched controls. In persons with a MACE, the apoB/apoA-1 ratio was elevated compared to controls already 20 years before the event and gradually increased during the 10 years closest to the event. The apoB/apoA-1 ratio increased initially also in the controls but levelled off, leaving a substantial gap between cases and controls. Similarly, apoB was higher in cases than in controls about 20 years before the event, while the apoA-1 curve separated later, with lower values for the cases about 10 years before the event.

In persons developing MI, the curves for the apoB/apoA-1 ratio separated already more than 20 years before the event, and the curve for cases increased gradually towards the time of event compared to the curve for controls. Similarly, apoB levels separated at more than 20 years before the event. ApoA-1 levels separated for cases versus controls about 10 years before the event and remained low.

Similar results were found in those undergoing CABG or PCI (S9 Supplement), but the difference in the apoB/apoA-1 ratio 20 years before the event was greater than for MACE and MI.

## Discussion

The present study is likely the largest so far designed to evaluate the association between the major transporters of lipids, i.e., apoB and apoA-1, and the apoB/apoA-1 ratio and MACE in a detailed way. The size of the cohort, the large number of events, and the long follow-up time allowed for a detailed assessment of the association. The major finding was a strong and graded relation between apoB, apoA-1, and the apoB/apoA-1 ratio and an increased risk of MACE and its sub-components. The higher the apoB level and the apoB/apoA-1 ratio, the higher was the risk. The results remained after adjusting for TC, TGs, glucose, sex, and SES, and after adjusting for smoking, diabetes, and hypertension as shown in a sensitivity analysis. All these associations were similar for men and women aged 25–69 years and somewhat weaker in both sexes for individuals between 70 and 84 years of age. In addition, similar results were seen across the sub-components of MACE—non-fatal MI, ischaemic stroke, and CV mortality—as well as for the coronary interventions CABG or PCI.

The rationale for using apoB and apoA-1 is that these surface proteins bind and transport cholesterol to and from peripheral tissues. The rationale for considering the balance between apoB and apoA-1, expressed as the apoB/apoA-1 ratio, is based on the pathophysiological role of apoB, which primarily reflects the number of small, dense, and most atherogenic LDL particles. These particles are formed in lipolytic processes from VLDL, IDL, and large buoyant LDL, eventually forming small, dense particles. The smallest cholesterol-containing particles can penetrate the arterial wall through the intima, where they deposit their cholesterol. The small apoA-1 protein can protect, reverse, and export cholesterol from the arterial wall back to the liver for further excretion through the bile into the gut. In addition to having this major anti-atherogenic role, the apoA-1 protein carries also protective cofactors that have anti-inflammatory, anti-oxidative, anti-thrombotic effects. ApoA-1 can also stimulate endothelial production of nitric oxide in the arterial wall, which has vasodilatory effects. Altogether this indicates that the apoB/apoA-1 ratio, reflecting the balance between atherogenic and anti-atherogenic particles, is a useful risk indicator for coronary heart disease and the development of atherosclerosis, in a similar manner as LDL-C and non-HDL-C or apoB only. Thus, a single value of the apoB/apoA-1 ratio can indicate the level of risk for multiple CV events.

An incidence proportion of MACE of 10% occurred about 8 years earlier in men and about 10 years earlier in women in the highest compared to the lowest apoB/apoA-1 ratio quintile. We also found using trajectories that those who had a MACE, MI, or coronary intervention had higher apoB/apoA-1 ratio and higher apoB than matched controls already about 20 years before the event. These differences increased over time and were greatest close to the event. These findings indicate that relatively young individuals have dyslipidaemia that may cause severe CV complications and thus should warrant early preventive measures. The burden of progressive atherosclerosis is dependent on both the age of onset of dyslipidaemia and the number of years this condition exposes the arterial wall.

We [10,14] and others [15–20] have previously reported that apoB has a strong relationship to LDL-C and that the apoB/apoA-1 ratio also has a strong relationship to the TC/HDL and LDL/HDL ratios and their association with risk of MI [9,30]. In the present ROC-AUC analyses of MACE and MI, we found that apoB had a somewhat stronger relationship to risk than LDL in women. The apoB/apoA-1 ratio also had a stronger relationship to MACE, CV death, and need for coronary interventions in women than men. Such data on risk for women at all ages have not, to our knowledge, previously been shown in such detail.

In the graphical ‘pyramid-like’ plots, increasing values of apoB and decreasing values of apoA-1, as expressed in the apoB/apoA-1 ratio, contribute to gradually increasing risk of MACE and MI. Of the 2 apolipoproteins, apoB had the stronger association with risk. However, a seemingly normal or low apoB value of 0.9 g/L or even 0.8 g/L might be associated with an increased CV risk in persons with low apoA-1 values, as reflected by the apoB/apoA-1 ratio. In this study a low apoA-1 potentiated the risk of MACE associated with apoB by about 40% on average. In patients with MI, this average increase was even higher (72%). Thus, it is not only the total concentration of apoB, but also the balance between apoB and apoA-1 expressed as the apoB/apoA-1 ratio that is strongly associated with CV risk. This apolipoprotein ratio also captures most types of dyslipidaemias including genetic forms [30,31].

In persons with hypertriglyceridaemia, type 2 diabetes, or metabolic syndrome and in obese persons, measurements of LDL-C are less accurate for methodological reasons, which is why non-HDL-C or apoB measurements are recommended [1–3,5,10]. The apoB/apoA-1 ratio is more strongly related to CV risks than apoB in persons with pre-diabetes and metabolic syndrome often characterised by hypertriglyceridaemia [30]. The apoB/apoA-1 ratio already about 20 years in advance is associated with development of type 2 diabetes, as seen in the AMORIS cohort [28]. The apoB/apoA-1 ratio is also associated with risk of MI and need for CABG or PCI in those who have these events before age 50 years [29].

Although recent international guidelines propose use of LDL-C and/or non-HDL-C as the primary risk variables, there is virtually no single study or clinical condition in which LDL-C or non-HDL-C or a lipid ratio has been shown to be superior to apoB or the apoB/apoA-1 ratio in predicting CV risk. Furthermore, few studies have shown better prediction by LDL-C, non-HDL-C, or apoB over the apoB/apoA-1 ratio for the amount of atherosclerosis based on calcium score, ultrasound measurements of the carotid and femoral artery, endothelial functions, or other CV measurements [10,32].

Can apolipoproteins also be used to monitor lipid-lowering treatments with statins and other lipid-lowering agents? Most lipid-lowering drugs, and especially statins, reduce both apoB and the apoB/apoA-1 ratio in a dose–response fashion, by about 20%–50%, and some also increase apoA-1 levels, but only by 5%–15% [33–37]. Importantly, in several large lipid-lowering trials the positive outcome of these trials has been more closely related to lowering of the apoB/apoA-1 ratio than lowering of LDL-C or any other lipid fractions [34–37].

Based on the results of the present large and comprehensive study, together with previous evidence, we propose that apoB should be included as a standard clinical test in general guidelines for early detection of persons at increased CV risk [1–10]. The apoB/apoA-1 ratio adds to risk information above apoB so that a broad range of persons at risk for clinical events can be identified early. Recommendations and appropriate cut-values should be agreed upon in new guidelines so that apoB, apoA-1, and especially the apoB/apoA-1 ratio can be used either as single or complementary risk markers for defining CV risk, and also as targets for lipid-lowering treatment.

The main strengths of this study include the large population of 137,100 individuals, comprising 57% men and 43% women and covering a wide age range from 25 to 84 years. The large number of incident MACEs (*n* = 22,473) is a major strength enabling detailed analysis. It is also a strength that all laboratory analyses were performed on fresh blood samples by the same laboratory, indicating stable and consistent quality of analytical conditions compared to large studies using meta-analysis based on many different older methods also applied on frozen samples stored for many years. Notably, apoB and apoA-1 are direct measurements of well-defined proteins independent of fasting conditions, with small errors compared with LDL-C, non-HDL-C, and lipid-based ratios [10,38]. The long follow-up time of about 20 years with basically no loss to follow-up of diagnosed cases enabled calculation of trajectories to define when in time a high apoB and apoB/apoA-1 ratio or a low apoA-1 value is seen before a CV event occurs. However, information about apolipoproteins was not available to us beyond the baseline period 1985–1996 and thus did not allow analyses of changes in biomarkers over time for individuals; rather, analyses were confined to population trajectories comparing cases and controls at different time points prior to the event.

There are several limitations to this study. We did not have access to all major CV risk factors for the full cohort, but in validation analysis we used information from 2 research cohorts with standardised protocols (WOLF and 60YO) performed in the Stockholm region during about the same period as the health examinations of the AMORIS cohort and with data on hypertension, self-reported diabetes, and tobacco smoking. The results from these analyses suggest little or no impact of adjustments for these additional risk factors on the association of the apoB/apoA-1 ratio and MACE.

For MACE we relied on routine diagnoses available in the National Patient Register and the National Cause of Death Register. Thus, a certain level of misclassification of the outcome is likely present but is probably unrelated to exposure, and therefore this misclassification should not substantially bias the HR estimates.

Another limitation is that no national Swedish register data on prescribed drugs were available until after June 2005. Most likely, some individuals were given CV and lipid-lowering drugs at baseline or during the study period that may have affected lipid, apolipoprotein, or glucose levels, probably leading in many cases to lower levels of the apoB/apoA-1 ratio during follow-up compared to baseline. This is likely to have, if anything, attenuated the observed associations and is unlikely to explain the observed associations between the apoB/apoA-1 ratio and MACE or its sub-components.

In conclusion, this large cohort study has shown that the apoB/apoA-1 ratio, which reflects the balance between atherogenic and athero-protective lipids, is a strong and graded long-term CV risk marker of MACE and its sub-components MI, stroke, and CV mortality, as well as coronary interventions, in both men and women of a wide age range. Differences in the apoB/apoA-1 ratio between future cases and controls were seen already about 20 years before these events occurred. Recommendations and appropriate cut-values should be agreed upon in new guidelines so that apoB, apoA-1, and especially the apoB/apoA-1 ratio may be used as markers for defining CV risk, and also as targets for lipid-lowering treatment.

## Supporting information

S1 Checklist STROBE Checklist. (DOCX)

S1 Data Estimates of unadjusted and adjusted hazard ratios. (XLSX)

S1 Supplement Additional information on materials and methods including definitions of BMI, co-morbidity, and ICD codes for cardiovascular diagnosis. (DOCX)

S2 Supplement Person-years at risk (PYR) and the number of events for selected outcomes in men and women by age group. (DOCX)

S3 Supplement Kaplan–Meier estimates for quintiles of the apoB/apoA-1 ratio for MACE for men in different age groups. (DOCX)

S4 Supplement Kaplan–Meier estimates for quintiles of the apoB/apoA-1 ratio for MACE for women in different age groups. (DOCX)

S5 Supplement Hazard ratios adjusted for TC, TGs, glucose, and SES for selected events, with 95% confidence intervals, for deciles of the apoB/apoA-1 ratio in men. (DOCX)

S6 Supplement Hazard ratios adjusted for TC, TGs, glucose, and SES for selected events, with 95% confidence intervals, for deciles of the apoB/apoA-1 ratio in women. (DOCX)

S7 Supplement Adjusted hazard ratios for combinations of deciles of apoA-1 and apoB in men and women combined. Outcomes: MACE (top left); myocardial infarction (middle left); ischaemic stroke (bottom left); cardiovascular mortality (top right); MACE, CABG or PCI (middle right); CABG or PCI (bottom right).
(DOCX)

S8 Supplement ROC-AUC values for apoB and LDL for MACE and myocardial infarction in men and women. *N* = 18,069.
(DOCX)

S9 Supplement Trajectories over a 25-year period prior to CABG or PCI for cases and controls in men and women combined. (DOCX)

## Abbreviations

60YO: Cohort of 60-year-olds
CABG: coronary artery bypass grafting
CV: cardiovascular
HDL: high-density lipoprotein
HDL-C: high-density lipoprotein cholesterol
HR: hazard ratio
IDL: intermediate-density lipoprotein
LDL: low-density lipoprotein
LDL-C: low-density lipoprotein cholesterol
MACE: major adverse cardiovascular event
MI: myocardial infarction
PCI: percutaneous coronary intervention
ROC-AUC: area under the receiver operating characteristic curve
SES: socioeconomic status
TC: total cholesterol
TG: triglyceride
VLDL: very-low-density lipoprotein
WOLF: Work, Lipids and Fibrinogen
