# Supplementary material for: Long-term risk of a major cardiovascular event by apoB, apoA-1, and the apoB/apoA-1 ratio—Experience from the Swedish AMORIS cohort: A cohort study
Source: PLoS Med. 2021 Dec 1;18(12):e1003853. doi: 10.1371/journal.pmed.1003853 (PMC8635349; doi:10.1371/journal.pmed.1003853)
Supplement: S1 Supplement — (DOCX) [file pmed.1003853.s003.docx]

**Supplement 1**

Additional facts on the analytical Methods

Validation of apolipoprotein data obtained in all years, which were analyzed by the same technique, was done in 1997 in collaboration with the Northwest Lipid Research Laboratories, University of Washington, Seattle, WA, USA. Correction factors of 1.059 for apoB and y=0·989x +0·101 for apoA-I were used to ensure that values fulfilled WHO-IFCC international criteria. The total coefficient of variation was below 3% for total cholesterol and below 5% for the other variables.

An extensive quality control scheme was used throughout the study. For measurement of apolipoproteins, daily samples from fresh or frozen pools of human sera were used as controls from the beginning of the study. The accuracy of total cholesterol and triglyceride values was checked against standards from the National Institute of Standards and Technology, Gaithersburg, MD, USA, or against analyses done at lipid reference laboratories certified by the US Centers for Disease Control and Prevention.

Four different populations—in which concentrations of lipids, apolipoproteins, and HDL- cholesterol were measured and LDL-cholesterol was calculated according to the Friedewald formula (6) were used to validate the newly developed formula, as follows: a subset of the AMORIS population (n=3861); individuals investigated after recruitment for AMORIS was completed (5430); a health check-up subset (512); and individuals having health screening (5761). In these four different populations, the correlation between concentration of LDL-cholesterol obtained by the formula based on total cholesterol (TC), triglycerides (TG), and apoA-I, and LDL-cholesterol calculated by Friedewald, was between r=0.97 and r=0.99. The newly derived formula did not systematically overestimate or underestimate low or high LDL-cholesterol concentrations (Bland-Altman plots). Therefore, the LDL-cholesterol concentrations calculated in our study population are valid. Furthermore, the level of correlation between apoB and LDL-cholesterol in our study and the analysed subsets (r=0.77–0.86) is of the same dimension recorded by the Friedewald formula in recent studies. These formulas have been validated by Talmud et al (8).

Information on BMI was available for 28 613 persons (21 %) from the baseline health examinations and in addition information on BMI was retrieved from other health examinations in AMORIS, the Swedish Medical Birth Register, from national quality of care registers, and from research cohorts previously linked to the AMORIS cohort (15). Information on history of cancer was retrieved from the National Cancer Register going back to 1958 and on kidney failure *and COPD* or asthma from the National Patient Register (NPR) going back to 1964 regionally, Stockholm County 1972 and nationally since 1987. The Charlson Comorbidity index was calculated based on information from the NPR.

ICD-codes

The following ICD codes that define each outcome are used: 1) Myocardial infarction: (ICD-9: 410, ICD-10: I21-I22), 2) Ischemic stroke, (ICD-9: 433-434, ICD-10: I63), 3) Haemorrhagic stroke: (ICD-9: 431, ICD-10: I61), 4) Cardiovascular mortality: (ICD-9: 390-459, ICD-10: I00-I99), MACE (sum of 1-4), CABG or PCI (codes for surgical interventions in Swedish registers defined in 1980: (3066, 3067, 3080, 3127) and the following codes defined since 1997 (FNA, FNB, FNC, FND, FNE, FNG).
