# Supplementary material for: Long-term risk of a major cardiovascular event by apoB, apoA-1, and the apoB/apoA-1 ratio—Experience from the Swedish AMORIS cohort: A cohort study
Source: PLoS Med. 2021 Dec 1;18(12):e1003853. doi: 10.1371/journal.pmed.1003853 (PMC8635349; doi:10.1371/journal.pmed.1003853)
Supplement: S2 Supplement — (DOCX) [file pmed.1003853.s004.docx]

**S2 Supplement**

Person-years under risk (PYR) and the number of events for selected outcomes in men and women by age group.

|  | **Men** | | | | |  | **Women** | | | | |
| --- | --- | --- | --- | --- | --- | --- | --- | --- | --- | --- | --- |
|  | **25-64^1^** | |  | **65-84^2^** | |  | **25-64^3^** | |  | **65-84^4^** | |
| **Type of event** | **PYR** | **Event** |  | **PYR** | **Event** |  | **PYR** | **Event** |  | **PYR** | **Event** |
| MACE | 1,320,196 | 11,287 |  | 57,973 | 2,701 |  | 958,106 | 4,538 |  | 105,890 | 3,947 |
| Myocardial infarction | 1,343,298 | 6,252 |  | 61,771 | 1,215 |  | 969,395 | 1,956 |  | 111,941 | 1,510 |
| Ischaemic stroke | 1,359,033 | 3,927 |  | 61,886 | 939 |  | 968,403 | 1,963 |  | 110,410 | 1,471 |
| Cardiovascular mortality | 1,382,577 | 4,004 |  | 65,867 | 1,888 |  | 979,492 | 1,616 |  | 116,602 | 2,856 |
| MACE, CABG or PCI | 1,299,645 | 13,270 |  | 56,044 | 2,819 |  | 953,988 | 4,960 |  | 105,005 | 4,003 |
| CABG or PCI | 1,338,239 | 5,800 |  | 62,362 | 416 |  | 970,503 | 1,293 |  | 114,557 | 268 |
