# Supplementary material for: Long-term risk of a major cardiovascular event by apoB, apoA-1, and the apoB/apoA-1 ratio—Experience from the Swedish AMORIS cohort: A cohort study
Source: PLoS Med. 2021 Dec 1;18(12):e1003853. doi: 10.1371/journal.pmed.1003853 (PMC8635349; doi:10.1371/journal.pmed.1003853)
Supplement: S3 Supplement — (DOCX) [file pmed.1003853.s005.docx]

| **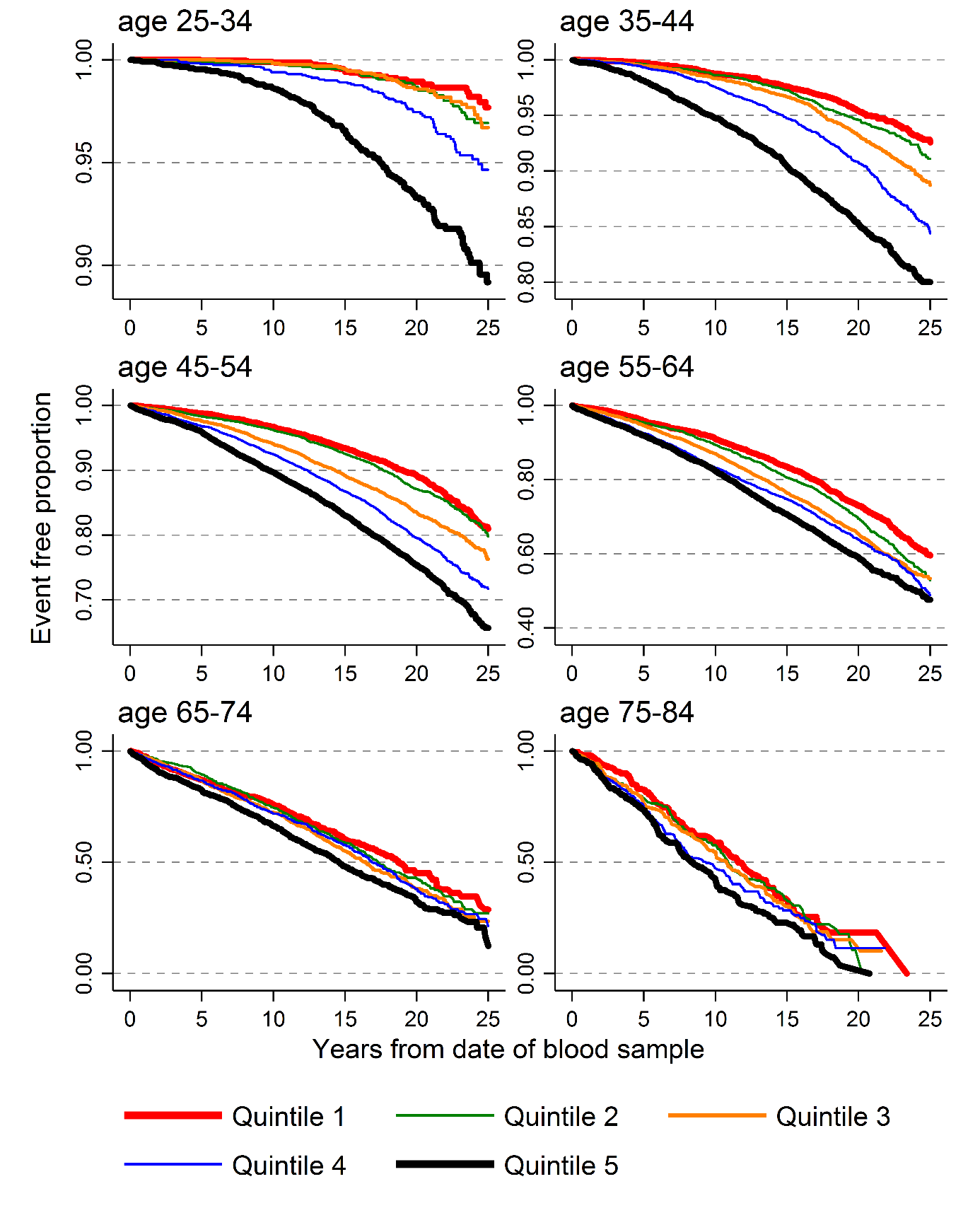** |
| --- |

**S3 Supplement.** Kaplan-Meier estimate for quintiles of the apoB/apoA-1 ratio for MACE for men in different age groups.
