# Supplementary material for: Long-term risk of a major cardiovascular event by apoB, apoA-1, and the apoB/apoA-1 ratio—Experience from the Swedish AMORIS cohort: A cohort study
Source: PLoS Med. 2021 Dec 1;18(12):e1003853. doi: 10.1371/journal.pmed.1003853 (PMC8635349; doi:10.1371/journal.pmed.1003853)
Supplement: S5 Supplement — (DOCX) [file pmed.1003853.s007.docx]

| **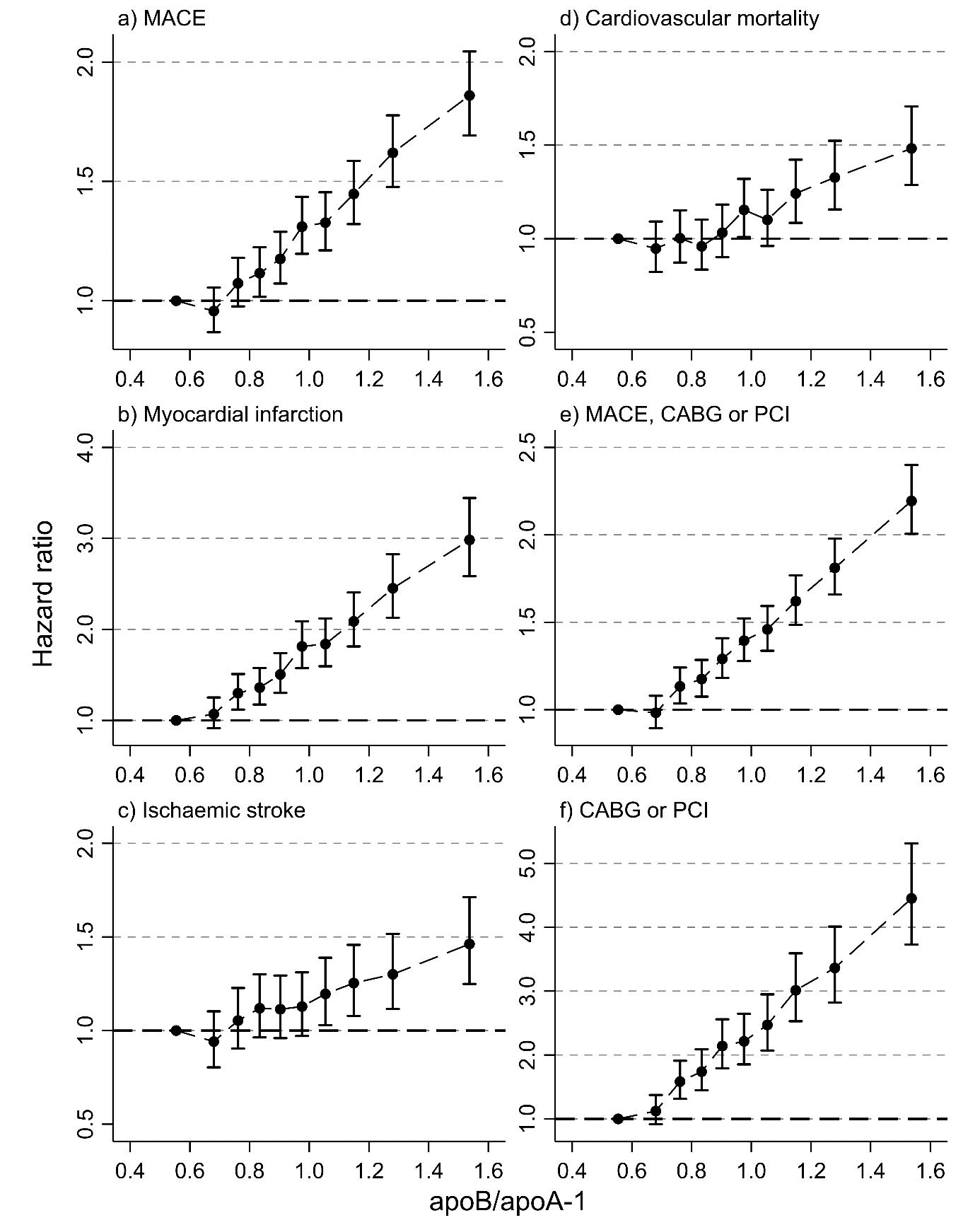** |
| --- |

**S5 Supplement**. Hazard ratio adjusted for TC, TG, glucose and SES for MACE, its subcomponents, and coronary interventions a) - f) with 95% confidence intervals for deciles of the apoB/apoA‑1 ratio in men.
