# Supplementary material for: Long-term risk of a major cardiovascular event by apoB, apoA-1, and the apoB/apoA-1 ratio—Experience from the Swedish AMORIS cohort: A cohort study
Source: PLoS Med. 2021 Dec 1;18(12):e1003853. doi: 10.1371/journal.pmed.1003853 (PMC8635349; doi:10.1371/journal.pmed.1003853)
Supplement: S7 Supplement — Outcomes: MACE (top left); myocardial infarction (middle left); ischaemic stroke (bottom left); cardiovascular mortality (top right); MACE, CABG or PCI (middle right); CABG or PCI (bottom right). (DOCX) [file pmed.1003853.s009.docx]

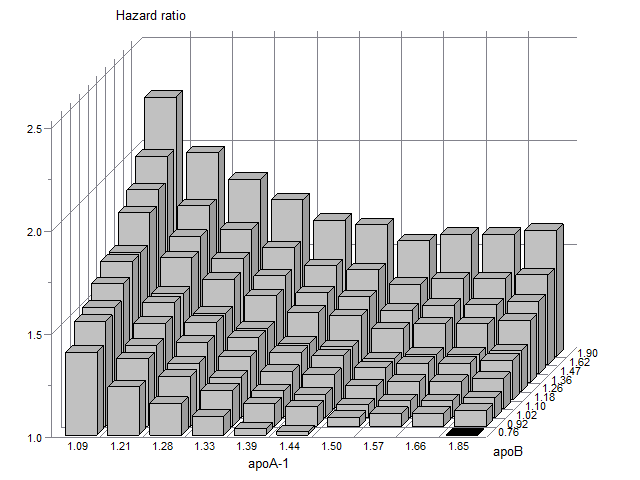

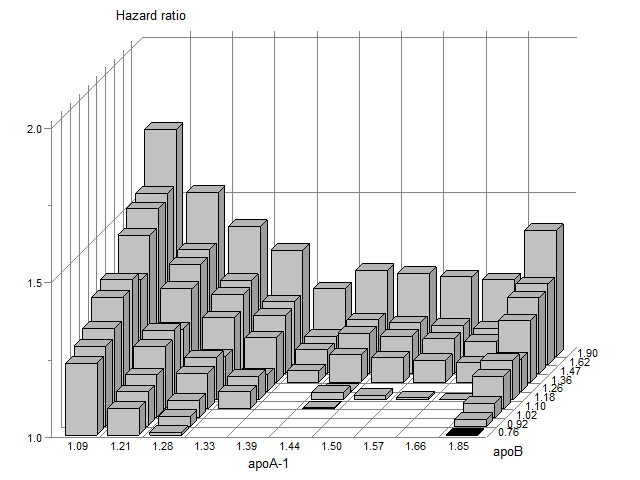


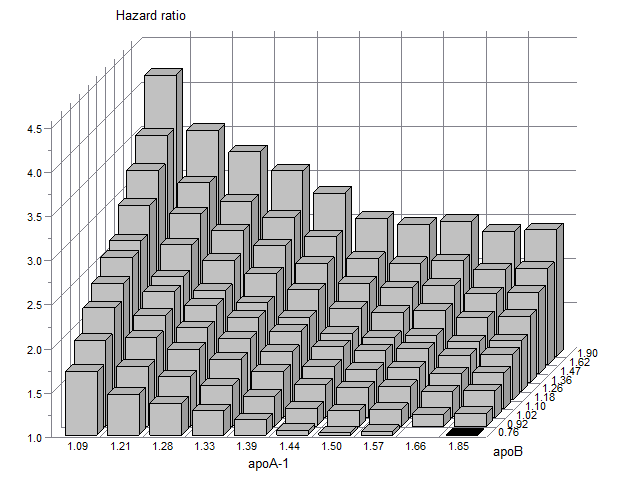

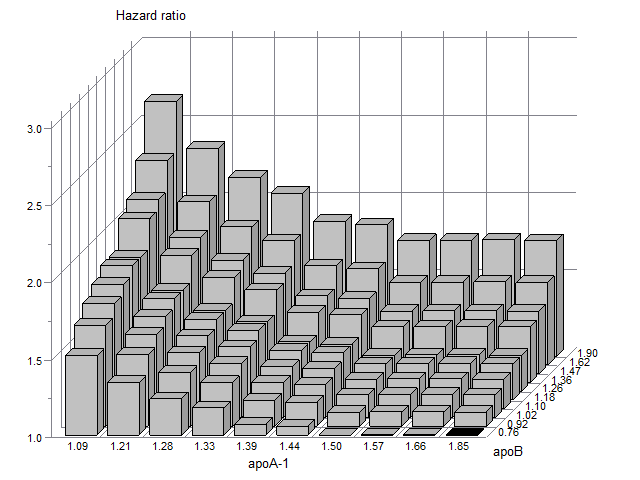


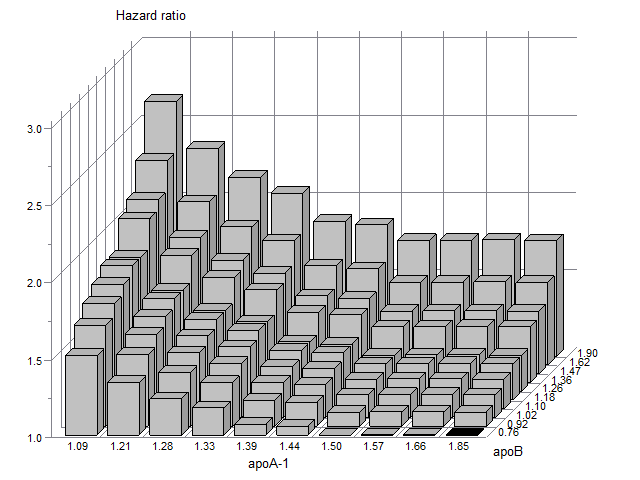

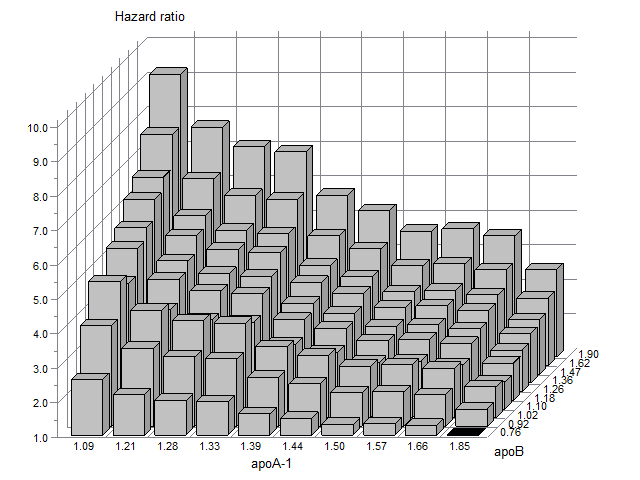


**Left column Right column**

**Top a), Middle b), Right c) Top d), Middle e), Right f)**

**S7 Supplement. H**azard ratio adjusted for TC, TG glucose, sex, and SES by the combination of deciles for apoA-1 and apoB. Men and women combined. a) MACE, b) Myocardial infarction, c) Ischaemic stroke, d) Cardiovascular mortality, e) MACE, CABG or PCI, and f) CABG or PCI.
