# Supplementary figures and images for: Long-term risk of a major cardiovascular event by apoB, apoA-1, and the apoB/apoA-1 ratio—Experience from the Swedish AMORIS cohort: A cohort study
Source: PLoS Med. 2021 Dec 1;18(12):e1003853. doi: 10.1371/journal.pmed.1003853 (PMC8635349; doi:10.1371/journal.pmed.1003853)

| **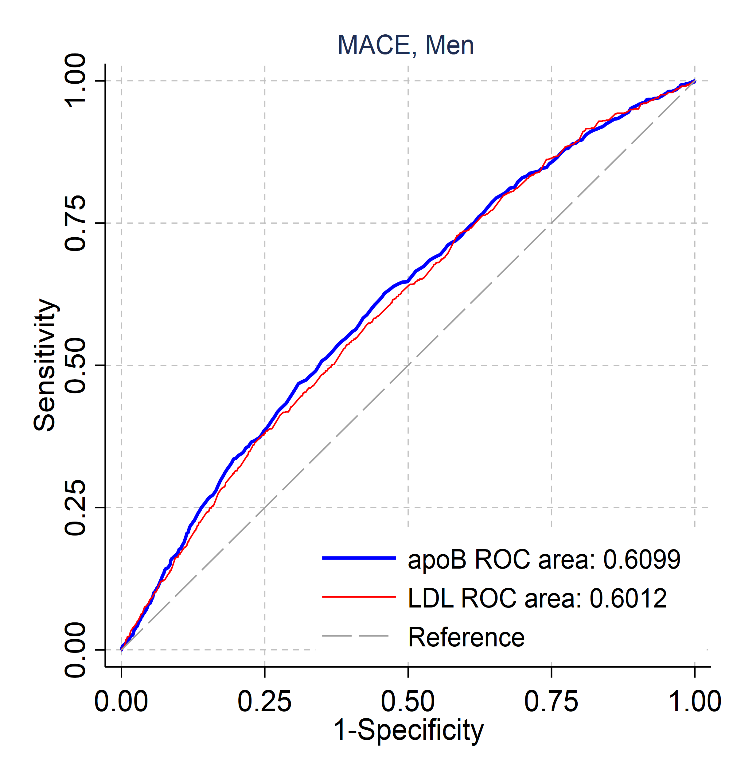** | **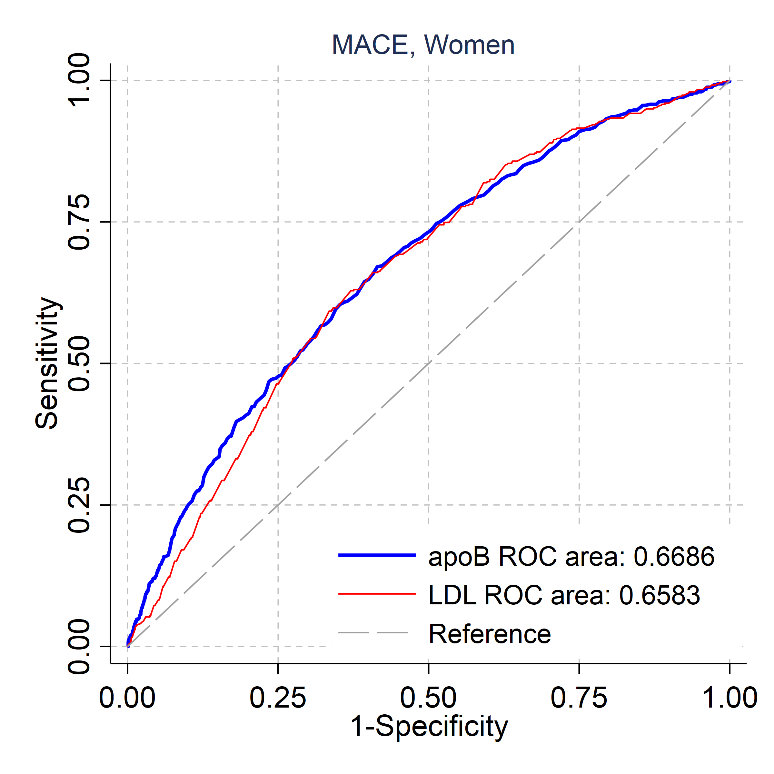** |
| --- | --- |
| **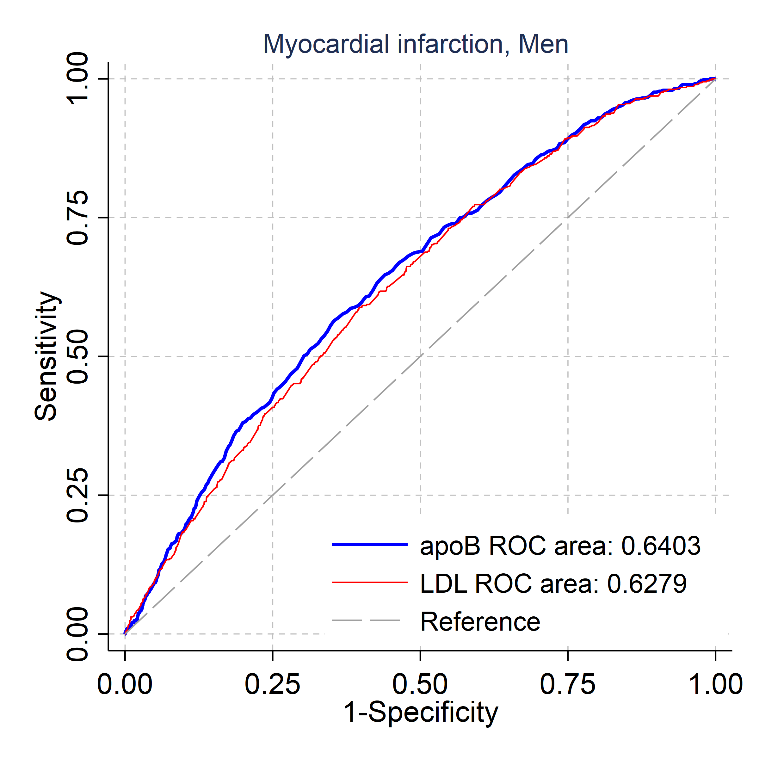** | **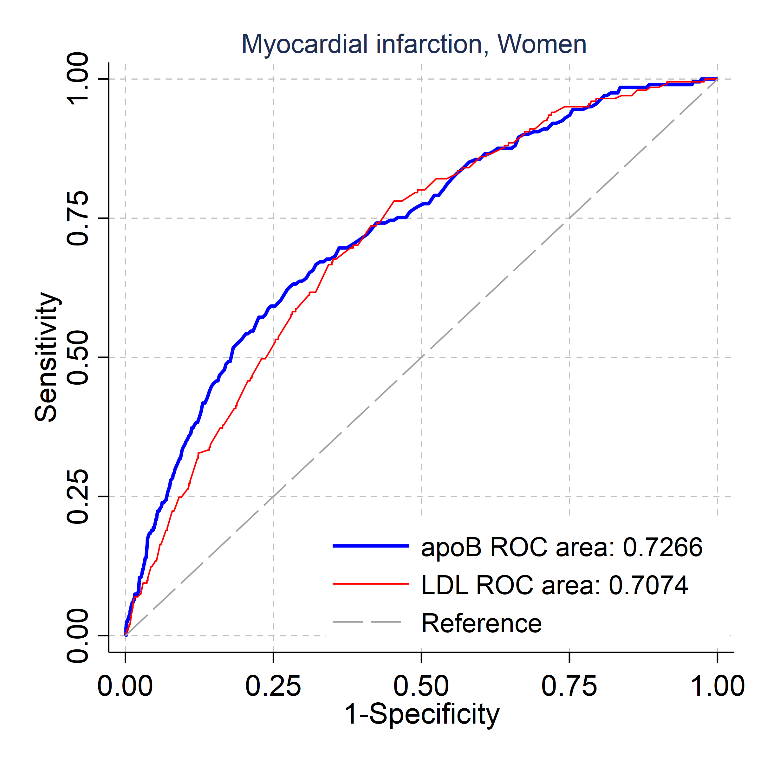** |

**S8 Supplement**. ROC-AUC areas for apoB and LDL for MACE and myocardial infarction in men and women. N = 18 069.

Supplement: S8 Supplement — N = 18,069. (DOCX) [file pmed.1003853.s010.docx]
