# Supplementary material for: Long-term risk of a major cardiovascular event by apoB, apoA-1, and the apoB/apoA-1 ratio—Experience from the Swedish AMORIS cohort: A cohort study
Source: PLoS Med. 2021 Dec 1;18(12):e1003853. doi: 10.1371/journal.pmed.1003853 (PMC8635349; doi:10.1371/journal.pmed.1003853)
Supplement: S9 Supplement — (DOCX) [file pmed.1003853.s011.docx]

| **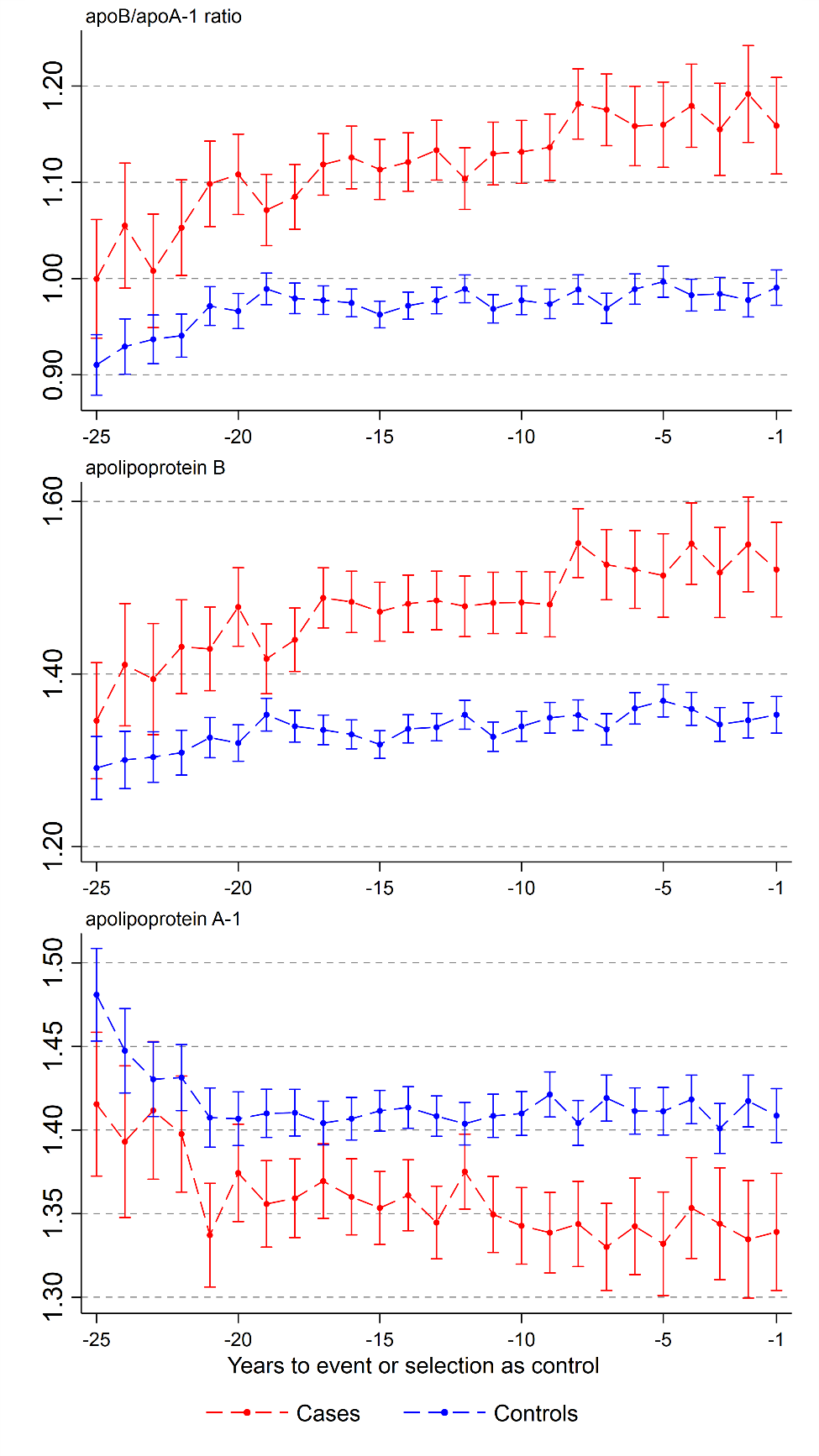** |
| --- |

**S9 Supplement .** Trajectories over a 25-year period prior to CABG/PCI for cases and controls, Men and women combined. Note the different scales on the levels of the apolipoproteins.
